# Protecting the primary care physicians' well‐being

**DOI:** 10.1002/jgf2.654

**Published:** 2023-10-30

**Authors:** Kiyoshi Shikino, Akemi Ando, Yutaro Okamoto, Asako Miyazawa, Taku Harada

**Affiliations:** ^1^ Department of General Medicine Chiba University Hospital Chiba Japan; ^2^ Department of Community‐Oriented Medical Education Chiba University Graduate School of Medicine Chiba Japan; ^3^ Ando Occupational Health Consultant Office Tokyo Japan; ^4^ Department of International Cooperation for Medical Education, International Research Center for Medical Education, Graduate School of Medicine The University of Tokyo Tokyo Japan; ^5^ Yamanashi City Makioka Hospital Yamanashi Japan; ^6^ Katsuta Hospital Hitachinaka Ibaraki Japan; ^7^ Department of General Medicine Nerima Hikarigaoka Hospital Tokyo Japan; ^8^ Department of Diagnostic and Generalist Medicine Dokkyo Medical University Hospital Shimotsuga‐gun Tochigi Japan

## Abstract

Seminar participants collaborated as a team to improve their organization, work environment, and labor issues using the Plan‐Do‐Check‐Act (PDCA) cycle. The PDCA cycle helps healthcare providers identify risks and hazards in their work environment and address daily issues. It guides them in planning and executing improvements while enabling progress tracking and encouraging further considerations for implementation.

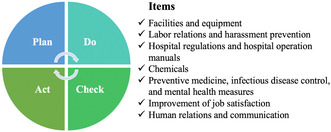

To the Editor:

In Japan, some studies have indicated that the quality of medical care declines or physician burnout increases as overwork and the number of shifts per week increase.^1^, ^2^ To improve patients' well‐being, the quality of medical care, and the provision of patient safety, physicians' well‐being is essential. In particular, Japan will introduce work‐style reforms in 2024.^3^

Physician well‐being awareness has grown, but research on defining the issue, identifying contributing factors, and offering effective solutions has taken decades to mature. Physician well‐being 2.0 emphasizes action and system‐based interventions targeting the root causes of occupational distress, shifting focus from individuals to systems, processes, teams, and leaders.^4^

The Family Medicine fellowship portfolio includes the theme of “caring for the healthcare providers themselves.” One of the learning objectives is to discuss initiatives to improve the well‐being of healthcare providers and their care teams. This newly established portfolio area in 2020 is still fully establishing its teaching and learning methods.

In July 2023, the online seminar “Well‐Being for Healthcare Providers ‐ Save them from Burnout” was held during the continuing professional development support seminar for Family Medicine fellows. The aims of the seminar were as follows:

1. understand well‐being, with a focus on well‐being 2.0;
2. identify factors contributing to physician burnout;
3. evaluate burnout using the Burnout Rating Scale;
4. identify strategies to prevent burnout; and
5. develop concrete plans to propose well‐being strategies for healthcare providers.

To meet these objectives, seminar participants collaborated as a team to improve their organization, work environment, and labor issues using the Plan‐Do‐Check‐Act (PDCA) cycle.^5^ The PDCA cycle helps healthcare providers identify risks and hazards in their work environment and address daily issues (Figure 1). It guides them in planning and executing improvements while enabling progress tracking and encouraging further considerations for implementation. Furthermore, the PDCA cycle is useful in reflecting the physician well‐being 2.0 concept to organizations from an occupational health perspective.

**FIGURE 1:**
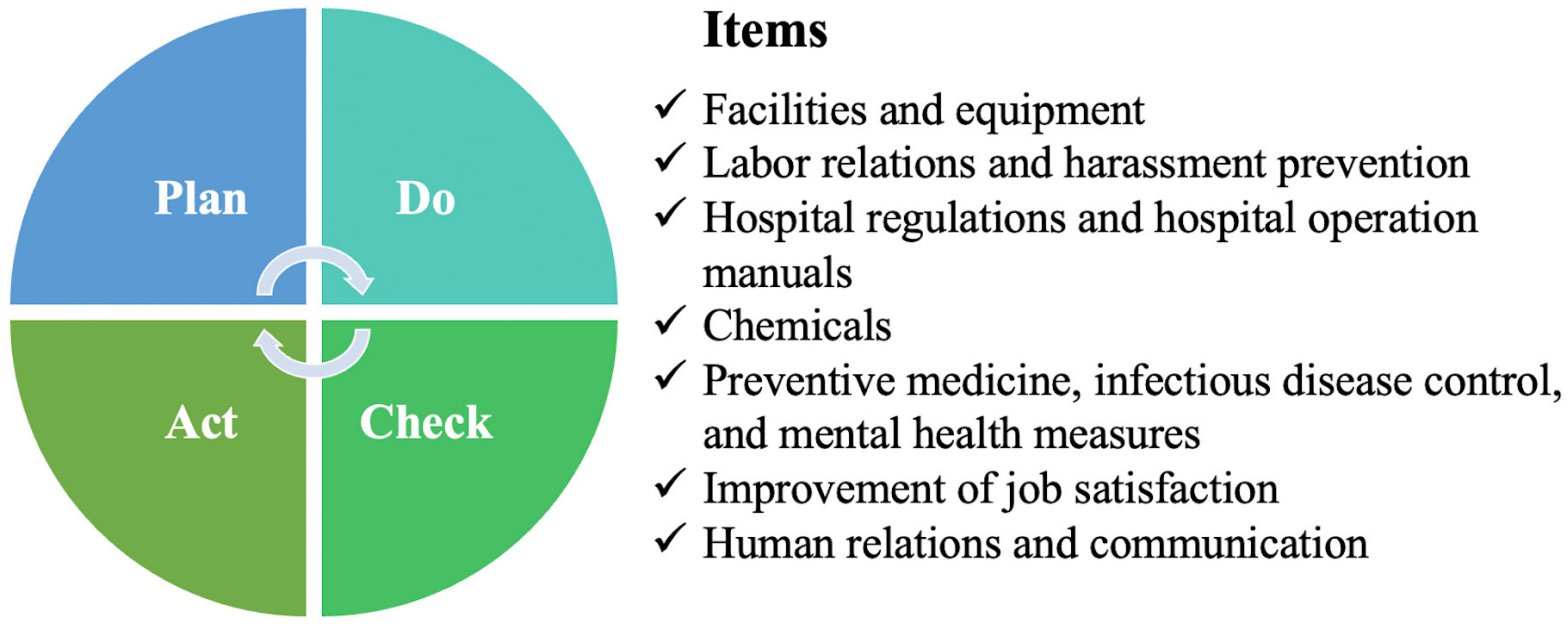
Plan‐Do‐Check‐Act cycle for improving health professionals' well‐being. The items represent specific elements extracted from the content used to enhance the working environment within medical institutions, particularly in the context of occupational health. The items serve as focal points for implementing the PDCA cycle to guide the continuous improvement efforts in accordance with the identified elements.

Forty‐seven participants (39 physicians and eight other healthcare providers, 64% response rate) responded to the 5‐point Likert scale questionnaire survey after the seminar. The scores were 4.2 ± 0.7 for “Understand what well‐being is,” 4.2 ± 0.6 for “List factors contributing to physician burnout,” suggesting a favorable outcome in terms of participant responses, supporting our argument.“ Other scores were 3.8 ± 0.7 for “Evaluate burnout using the Burnout Rating Scale,” 3.9 ± 0.6 for “Identify strategies to prevent burnout,” and 3.6 ± 0.6 for “Develop concrete plans to propose well‐being strategies for healthcare providers.”

Understanding how to prevent burnout will significantly impact the future well‐being of primary healthcare providers. We hope this framework will guide us in implementing measures to improve workplace well‐being.

## CONFLICT OF INTEREST STATEMENT

None.

## References

[1] Nonaka S , Makiishi T , Nishimura Y , Nagasaki K , Shikino K , Izumiya M , et al. Prevalence of burnout among internal medicine and primary care physicians before and during the COVID‐19 pandemic in Japan. Intern Med. 2022;61(5):647–651. 34924459 10.2169/internalmedicine.8118-21 PMC8943365

[2] Shikino K , Kuriyama A , Sadohara M , Matsuo T , Nagasaki K , Nishimura Y , et al. Work‐related stress and coping methods of internists and primary care physicians during the COVID‐19 pandemic in Japan: a mixed‐method study. J Gen Fam Med. 2022;23(5):327–335. 35942469 10.1002/jgf2.560 PMC9347816

[3] Takagi K , Tagami T . Work‐style reform of emergency physicians: the Japanese experience. Eur J Emerg Med. 2019;26(6):398–399. 31688220 10.1097/MEJ.0000000000000640

[4] Shanafelt TD . Physician well‐being 2.0: where are we and where are we going? Mayo Clin Proc. 2021;96(10):2682–2693. 34607637 10.1016/j.mayocp.2021.06.005

[5] Nino V , Claudio D , Valladares L , Harris S . An enhanced kaizen event in a sterile processing department of a rural hospital: a case study. Int J Environ Res Public Health. 2020;17(23):8748. 33255618 10.3390/ijerph17238748 PMC7728093

